# Better Together: Momentary Physiological Benefits of Co-Experienced Positive Emotions in Late Life

**DOI:** 10.1093/geroni/igaf122.2104

**Published:** 2025-12-31

**Authors:** Tomiko Yoneda, Nathan Lewis, Theresa Pauly, NIlam Ram, Denis Gerstorf, Claudia Haase, Christiane Hoppmann

**Affiliations:** University of California Davis, Davis, California, United States; The University of British Columbia, Vancouver, British Columbia, Canada; Simon Fraser University, Vancouver, British Columbia, Canada; Stanford University, Stanford University, California, United States; Humboldt University Berlin, Humboldt University Berlin, Berlin, Germany; Northwestern University, Evanston, Illinois, United States; UBC, Vancouver, British Columbia, Canada

## Abstract

Although positive emotions tend to be experienced most frequently and intensely within social interactions, most research has focused on positive emotions at the individual level. Yet, emerging research shows that shared positive emotions predict health and longevity over and above individually experienced positive emotions. However, the extent to which relationship partners co-experience positive emotions in daily life remains poorly understood. Moreover, whether positive emotions “get under the skin,” shaping proximal biomarkers in everyday life, is unclear. Drawing on Positivity Resonance Theory, we examined the links between co-experienced positive emotions and momentary cortisol levels in 321 older couples (ages 56–89). Findings showed that both relationship partners reported higher-than-usual positive emotions during 38% of the occasions they were together. Additionally, co-experienced positive emotions were linked to lower cortisol levels at the same occasion, adjusting for individually experienced positive emotions and several additional individual-level and time-varying covariates. Findings did not differ across age, sex, or relationship satisfaction, suggesting that the physiological benefits of co-experienced positive emotions are not dependent on individual or relationship characteristics. Notably, co-experienced positive emotions were also associated with lower cortisol at the subsequent assessment (but not vice versa), indicating that the benefits of co-experienced positive emotions extend beyond the immediate moment. This study highlights the important role that shared positive emotions play in promoting physiological health in older adults, providing evidence that co-experienced emotions offer benefits beyond individual experiences.

